# Sensorimotor Gating in Depressed and Euthymic Patients with Bipolar Disorder: Analysis on Prepulse Inhibition of Acoustic Startle Response Stratified by Gender and State

**DOI:** 10.3389/fpsyt.2018.00123

**Published:** 2018-04-18

**Authors:** Junko Matsuo, Miho Ota, Shinsuke Hidese, Toshiya Teraishi, Hiroaki Hori, Ikki Ishida, Moeko Hiraishi, Hiroshi Kunugi

**Affiliations:** Department of Mental Disorder Research, National Institute of Neuroscience, National Center of Neurology and Psychiatry, Tokyo, Japan

**Keywords:** prepulse inhibition, bipolar disorder, habituation, depression, euthymic, gender difference, psychosis

## Abstract

**Background:**

Prepulse inhibition (PPI) of the acoustic startle reflex is an operational measure of sensorimotor gating. The findings on PPI deficits in bipolar disorder (BD) are inconsistent among studies due to various confounding factors such as gender. This study aimed to assess sensorimotor gating deficits in patients with BD stratified by gender and state (depressed/euthymic), and to explore related clinical variables.

**Methods:**

Subjects were 106 non-manic BD patients (26 BD I and 80 BD II; 63 with depression and 43 euthymic) and 232 age-, gender-, and ethnicity-matched (Japanese) healthy controls. Depression severity was assessed using the Hamilton Depression Rating Scale-21. The electromyographic activity of the orbicularis oculi muscle was measured by a computerized startle reflex test unit. Startle magnitude, habituation, and PPI were compared among the three clinical groups: depressed BD, euthymic BD, and healthy controls. In a second analysis, patients were divided into four groups using the quartile PPI levels of controls of each gender, and a ratio of the low-PPI group (<1st quartile of controls) was compared. Effects of psychosis and medication status were examined by the Mann–Whitney *U* test. Clinical correlates such as medication dosage and depression severity with startle measurements were examined by Spearman’s correlation.

**Results:**

Male patients with depression, but not euthymic male patients, showed significantly lower PPI at a prepulse of 86 dB and 120 ms lead interval than did male controls. More than half of the male patients with depression showed low-PPI. In contrast, PPI in female patients did not differ from that in female controls in either the depressed or euthymic state. Female patients with active psychosis showed significantly lower PPI than those without psychosis. Female patients on typical antipsychotics had significantly lower PPI, than those without such medication. PPI showed a significant positive correlation with lamotrigine dosage in male patients and lithium dosage in female patients.

**Conclusion:**

These findings suggest that sensorimotor gating is impaired in male BD patients with depression. However, we obtained no evidence for such abnormalities in female BD patients except for those with current psychosis. The observed associations between medication and startle measurements warrant further investigation.

## Introduction

Prepulse inhibition (PPI), an operational measure of sensorimotor gating, is defined as the attenuation of the startle reflex when the startle-eliciting stimulus—the pulse—is preceded by a weaker sensory stimulus—the prepulse (PP) (1). It is typically measured by electromyographic (EMG) recordings from the orbicularis oculi muscle and is a very robust function; it is conserved across many species (2). PPI deficits have been associated with multiple neuropsychiatric disorders characterized by inhibitory deficits in sensory, motor, and cognitive functions, including schizophrenia, psychotic mania, obsessive–compulsive disorder, and Tourette syndrome (3, 4). Our group demonstrated PPI deficits in Asian individuals with schizophrenia for the first time (5), and subsequently reported that PPI was impaired in female and male patients with schizophrenia using a large sample of single ethnicity patients and healthy individuals (Japanese) (6). Brain imaging studies have revealed common abnormalities in the cortico–striato–pallido–pontine and cortico–striato–pallido–thalamic (CSPT) circuitries across these disorders; these circuits are considered to affect disease pathophysiology and PPI modulation (3, 7). Accumulating evidence from animal and human studies indicates that PPI is also modulated by top-down, higher-order cognitive regions, such as the hippocampus, medial prefrontal cortex (mPFC), orbitofrontal cortex (OFC), and basolateral amygdala (4, 8, 9). The amygdala, mPFC, and OFC, as well as the anterior cingulate gyrus and insula, have been consistently shown to be associated with emotional processing (10–12). These nuclei are dense in noradrenergic receptors and critical for the regulation of the hypothalamic–pituitary–adrenal and hypothalamic–pituitary–gonadal axes (13). Pharmacological studies in animal models of schizophrenia have also suggested the involvement of several neurotransmitter pathways, including dopaminergic, serotonergic, glutamatergic, and cholinergic pathways, in PPI deficiency (3, 14).

Prepulse inhibition deficits in patients with schizophrenia have been reported to be associated with positive symptoms (6, 15–18), negative symptoms (15, 18), disorganization symptoms (6), distractibility (19), thought disorder (20, 21), formal thought disorder and bizarre behavior (22), psychological discomfort (23), and general psychopathology (18, 24). A longitudinal study on patients with schizophrenia reported that PPI deficits in medicated patients were observed in acute illness, but not in an improved clinical state, which suggests that PPI deficits may be state dependent (22).

While PPI deficits in schizophrenia have been reported in many studies (3), little is known about gating differences in patients with BD (4), despite the occurrence of psychotic symptoms and cognitive impairments similar to schizophrenia patients (25, 26). Moreover, larger brain ventricle volumes and structural abnormalities in CSPT circuits have been detected in BD patients (27, 28). Genome-wide association studies (GWAS), human postmortem brain studies, and magnetic resonance spectroscopy studies have identified similar gene expression changes in glutamatergic neurotransmission and glutamate receptor (GluR) expression among patients with schizophrenia, BD, and major depressive disorder (MDD) (29–32). Since the glutamatergic *N*-methyl-d-aspartate receptors (NMDA) receptor antagonist, such as ketamine and phencyclidine, disrupt PPI (3), it is possible that the PPI may be impaired in BD. However, to our knowledge, there are only two studies that examined PPI in BD patients in manic states, and five studies in euthymic states, both with inconsistent findings. Perry et al. reported that PPI and habituation were exacerbated in acute psychotic mania, suggesting a possible association between PPI deficits, psychosis, and thought disturbance (33). A subsequent study by Carroll et al. failed to replicate these findings in manic and mixed episodic patients. Since almost all the patients in the latter study did not have psychosis (with only 1 exception), they concluded that acute psychosis might be necessary for the occurrence of BD-associated PPI deficits (34). In euthymic patients with BD, controversial results of both unimpaired (35, 36) and significantly lower PPI (37, 38) have been reported. Giakoumaki’s study also found reduced PPI in unaffected siblings of BD patients, suggesting that such disruption may represent a trait deficit in BD. All the above studies were conducted with men and women included in the same group, although gender-related differences in PPI (men > women) have been well-replicated in healthy subjects (3, 39–42). It is known that women present fluctuations in PPI across the menstrual cycle, with the lowest PPI in the mid-luteal phase when ovarian hormones (estrogen and progesterone) are maximal (41, 43, 44). Thus, Gogos et al. examined PPI in euthymic patients with BD stratified by gender and reported sexually dimorphic differences: male patients showed reduced PPI, while female patients in the follicular phase had increased PPI compared to their healthy counterparts (45). No study has yet assessed the PPI in BD patients with depression and the association of PPI with depression severity. Thus, it is still unclear whether PPI deficits represent a state or trait feature of BD.

Prepulse inhibition in patients with MDD was generally considered unimpaired (46–48); however, Perry et al. (46) observed moderate effect size of difference (Cohen’s *d* = 0.63) between patients with severe MDD and healthy controls. MRI examination of MDD patients revealed abnormalities in CSPT circuitry similar to those observed in patients with schizophrenia and BD (49–52), and two recent studies have found PPI deficits in MDD patients. One is ours reporting a significant negative correlation between PPI and depression severity in male, but not female, patients, suggesting that PPI impairment is state- rather than trait-dependence in male patients with MDD (53). The other study found PPI deficits in women with postpartum depression compared to their non-depression counterparts, when effects of ovarian hormones were minimal in all subjects (54).

In addition to gender, there are other factors that may influence PPI, such as age (55–57), ethnicity (58), and smoking status (59–62). Ethnic differences in startle magnitude and PPI were reported between Caucasians and Asians, with Asians having lower startle magnitude and higher PPI compared to Caucasians (58, 63, 64). To control for these confounding factors, we matched for age, gender, smoking status, and ethnicity.

In this study, we examined the modulation of the startle reflex in non-manic BD patients with the following aims: (1) to investigate whether BD patients with depression show deficits in PPI, startle reactivity, and habituation compared to euthymic patients and healthy individuals, and (2) to examine whether such deficiencies, if any, are associated with symptoms and other clinical features. Data acquired from men and women were analyzed separately. Based on our previous findings of reduced PPI in male patients with MDD, we hypothesized that depressed patients with BD, especially men, may also present PPI deficits. Additionally, we hypothesized that PPI deficits, if any, may be associated with depression severity, the presence of current psychosis, and more severe psychopathology.

## Materials and Methods

### Subjects

Subjects included 338 volunteers, consisting of 106 non-manic BD patients (26 BD I and 80 BD II; 63 patients with depression and 43 euthymic patients, see definition below) and 232 healthy individuals (age: 18–64 years). Data from control subjects were age-, gender-, and ethnicity-matched (Japanese) with those acquired from BD patients. Participants were recruited for neurocognitive research studies between 2009 and 2017 at the National Center of Neurology and Psychiatry (NCNP), Tokyo, Japan, through notices posted in the NCNP Hospital, website announcements, and advertisements in a local free paper. Most healthy individuals used as controls overlapped with those from our previous studies (6, 53). The participants were either NCNP Hospital inpatients (14%) or outpatients from the NCNP Hospital or other local hospitals and clinics.

All subjects were interviewed by experienced psychiatrists using the Japanese version of the Mini-International Neuropsychiatric Interview (65, 66). Diagnoses were further confirmed through medical records and detailed interviews based on the Diagnostic and Statistical Manual (DSM) of Mental Disorders, Fourth Edition, text revision (DSM-IV-TR) (67). Individuals with a concurrent confirmed diagnosis of intellectual disability or organic brain disorder, ongoing thyroid gland malfunction, undergoing electroconvulsive therapy treatment or substance abuse history in the previous year were excluded from this study. Patients with concurrent psychiatric disorders, such as anxiety disorder, panic disorder, autism spectrum disorder (ASD), or attention-deficit hyperactivity disorder, were included in this study. Control subjects with a psychiatric history or family history of mental illness within second-degree relatives (schizophrenia, BD, and ASD) were excluded from this study. None of the subjects presented with hearing deficits as confirmed by audiometry (threshold: average hearing level of 500, 1,000, and 2,000 Hz to <40 dB). Premorbid intelligence quotient (IQ) was estimated from the Japanese Adult Reading Test scores (68) and only individuals with premorbid IQ ≥ 85 were included in this study.

The depression severity of subjects with BD was assessed using the 21-item version of the Hamilton Depression Rating Scale (HAM-D21) (69). Manic symptoms of subjects with BD were assessed by the Young Mania Rating Scale (YMRS) (70). Based on the definition of manic states, as determined by the International Society for Bipolar Disorders Task Force (71), those in a significant manic, hypomanic, or mixed state (i.e., YMRS score ≥ 8) were excluded. Subjects with BD were further categorized into either the depressed (HAM-D17 ≥ 8 and YMRS < 8) or euthymic (HAM-D17 < 8 and YMRS < 8) group, according to the consensus definition for remission (72). A daily dose of antidepressants was calculated as imipramine equivalents, and antipsychotics as chlorpromazine equivalents in milligrams/day according to the published guidelines (73).

This study was conducted following the latest version of the Declaration of Helsinki. The study design was reviewed and approved by the NCNP Ethics Committee. Written informed consent for participation in this study was obtained from every subject after the nature of the procedures had been fully explained.

### Startle Reflex Measurement

#### PPI Paradigm

The EMG activity of the orbicularis oculi muscle was measured by a computerized startle reflex test unit. All participants were requested to refrain from smoking at least 30 min prior to testing, based on a previous study reporting that the PPI-enhancing effect of smoking lasts only for a short period (less than 10 min) (61). The apparatus, procedures, stimuli, and PPI paradigm used have been described in detail elsewhere (5, 53). Briefly, each session consisted of three blocks with 70 dB background noise. Blocks 1 and 3 consisted of additional 115 dB pulse alone (PA; five times each) trials. Block 2 was a pseudo-randomized combination of the same PA together with PP trials under four conditions (lead interval, intensity: 60 ms, 86 dB; 60 ms, 90 dB; 120 ms, 86 dB; 120 ms, 90 dB; five times each). In total, 35 trials of startle reflex were carried out in one session, lasting for 15 min.

#### Outcome Measures and Data Reduction

Outcome measures for analysis were as follows: (1) mean PA startle reflex magnitude (digital unit) in block 1, defined as basic startle reflex (BSR), (2) startle reflex habituation (%), and (3) PPI (%) for each PP condition. Mean PPI calculation and habituation were performed as described elsewhere (5, 53). Non-responding subjects were excluded from any further analysis (*n* = 45; BSR < 0.05 digital unit). Therefore, viable habituation and PPI data were collected from 87 BD patients and 206 healthy controls (total *n* = 293).

### Statistical Analysis

Statistical analyses were performed using SPSS Version 22.0 (SPSS Japan, Tokyo). Groups were compared based on demographic and clinical characteristics using independent Student’s *t*-tests or one-way analysis of variance for continuous variables and chi-squared test for categorical variables. Data from the left eye were selected for analysis because no sidedness was detected in any startle reflex measurements. According to the Shapiro–Wilk test, data from all startle measurements were not normally distributed (all *p* < 0.001); therefore, non-parametrical analyses were applied to these variables. We compared the startle measurements of three clinical groups (i.e., depressed BD, euthymic BD, and healthy control) with the Kruskal–Wallis test, followed by between-group comparisons with the Mann–Whitney *U* test. The findings were confirmed when patients with concurrent psychiatric disorders, such as anxiety disorder, panic disorder, ASD, and/or attention-deficit hyperactivity disorder, were excluded; therefore, they were retained in the analysis. Subjects were further subcategorized into four groups using the quartile PPI_120ms_86dB_ levels of controls for each gender. Then, the incidence of the low-PPI (first quartile group) vs. high-PPI (second to fourth quartile groups) was compared between patients and controls by the chi-squared test and across the three clinical groups by the Fisher’s exact test. Effects of active psychosis and medication status on habituation and PPI were examined by the Mann–Whitney *U* test. Spearman’s rank correlation coefficients of habituation and PPI percentage with clinical variables were computed. Statistical significance was set at a two-tailed *p* < 0.05.

## Results

**Figure 1:**
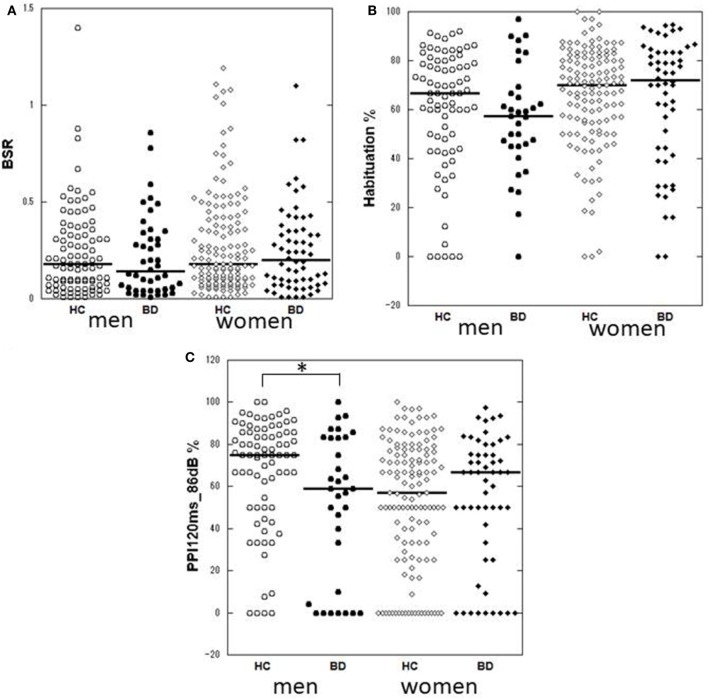
Dot-plots of startle measurements of the two diagnostic groups by gender. **(A)** Basic startle reflex (BSR) in block 1. **(B)** Habituation percentage. **(C)** Prepulse inhibition (PPI) percentage at a prepulse of 120 ms at 86 dB. Bar indicates median. Habituation and PPI < 0% are shown as 0%. Between-group differences were examined by the Mann–Whitney *U* test. *p < 0.05 uncorrected. HC, healthy control; BD, bipolar disorder.

**Figure 2:**
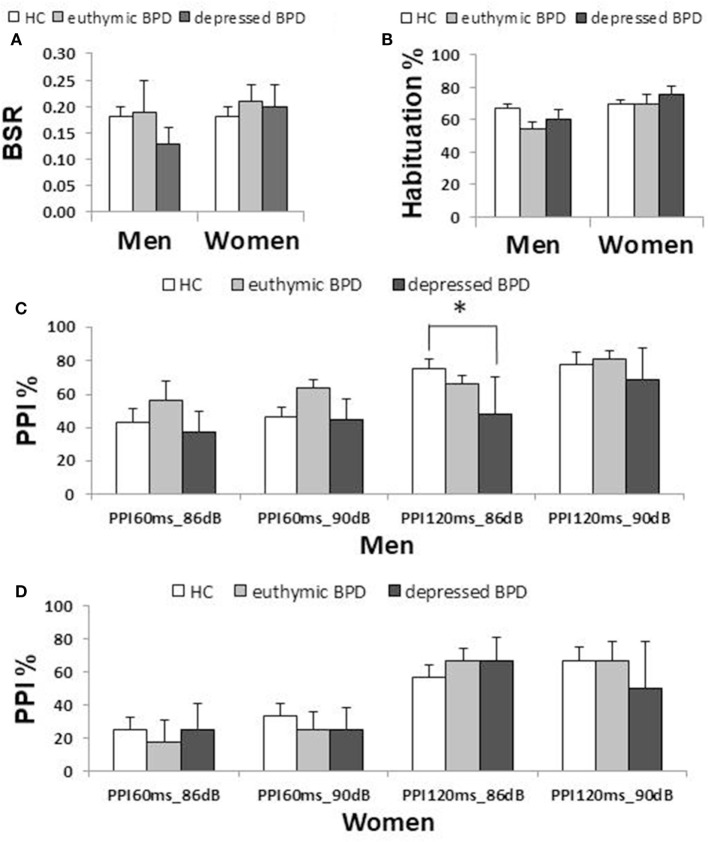
Comparison of startle measurements across the three clinical groups by gender (median ± SEM). **(A)** Basic startle reflex (BSR) in block 1. **(B)** Habituation percentage. **(C)** Prepulse inhibition (PPI) percentage in different prepulse parameter trials in men. **(D)** PPI percentage in women. Bar indicates median. Error bar indicates SEM. Between-group differences were examined by the Mann–Whitney *U* test. *p < 0.05 corrected. HC, healthy control; BD, bipolar disorder.

**Figure 3:**
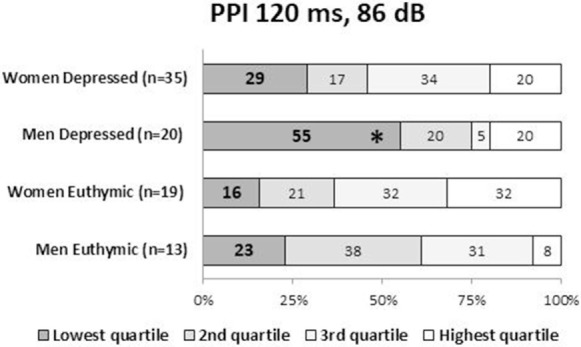
Ratio (%) of the PPI quartile groups in depressed and euthymic patients by gender. Patients were categorized into four groups using the PPI quartiles of healthy men and women. The incidence of the low-PPI (lowest quartile group) vs. high-PPI (second to fourth quartile groups) was compared across the three clinical groups by the Fisher’s exact test. Statistical significance was set at a two-tailed *p* < 0.05. *p < 0.05 against controls. PPI, prepulse inhibition.

Demographic and clinical characteristics of the total subjects are presented in Table 1. Because of the *a priori* matching, the two diagnostic groups were similar concerning gender and age distribution. Education years, current smoker ratio, and estimated premorbid IQ were not significantly different between the diagnostic groups. However, when non-responders were excluded, age and premorbid IQ were significantly higher in male controls than male patients [*t*(77.1) = 2.018, *p* = 0.047 and *t*(104) = 2.068, *p* = 0.041, respectively] (Table S1 in Supplementary Material). Depression severity in responders was not significantly different between male and female patients either in the total [*t*(56.2) = 0.667, *p* = 0.508], depressed [*t*(53) = 1.560, *p* = 0.125] or euthymic group [*t*(30) = −0.543, *p* = 0.591]. Comparisons of startle reflex responses between the diagnostic groups and across the three clinical groups are shown in Figures 1 and 2, respectively, as well as in Table S2 in Supplementary Material. The same comparison was made excluding patients with concurrent psychiatric disorders, and the results are provided in Table S3 in Supplementary Material. The ratio of the number of individuals in the PPI quartile groups of depressed and euthymic patients is shown in Figure 3. Effects of active psychosis and medication status, and correlation of clinical variables with habituation and PPI are presented in Tables 2 and 3, respectively.

**Table 1:** Demographic and clinical data of the subjects stratified by gender (mean ± SD).

	Bipolar disorder			Healthy controls				Statistical comparison	
	Total	Men	Women	Total	Men	Women	Total	Men	Women
Gender ratio, *N* (%)^a^	106	44 (42%)	62 (58%)	232	93 (40%)	139 (60%)	*x*^2^(l) = 0.061, *p* = 0.805	–	–
Age (years)	39.3 ± 10.0	40.5 ± 9.9	38.4 ± 10.2	41.6 ± 12.2	43.0 ± 13.1	40.7 ± 11.6	*t*(244.4) = 1.863, *p* = 0.064	*t*(109.1) = 1.232, *p* = 0.220	*t*(199) = 1.362, *p* = 0.175
Education (years)	15.1 ± 2.4	15.3 ± 2.1	14.6 ± 2.0	14.9 ± 2.1	15.7 ± 2.8	14.6 ± 2.1	*t*(336) = −0.739, *p* = 0.460	*t*(135) = −1.056, *p* = 0.293	*t*(199) = 0.018, *p* = 0.985
Current smoker, *N* (%)^a^^,^^b^	23 (22%)	14 (33%)	9 (15%)	46 (20%)	24 (26%)	22 (16%)	*x*^2^(l) = 0.179, *p* = 0.672	*x*^2^(l) = 0.550, *p* = 0.458	*x*^2^(l) = 0.045, *p* = 0.831
Premorbid IQ^c^	112 ± 9	112 ± 9	112 ± 8	113 ± 7	114 ± 7	112 ± 7	*t*(330) = 0.530, *p* = 0.597	*t*(131) = 1.708, *p* = 0.090	*t*(197) = −0.764, *p* = 0.446
Range	85–126	88–125	85–126	92–127	92–127	93–124			

**Clinical variables**	**Total**	**Men**	**Women**	**Men vs. women**					
Bipolar LN (%)^a^	26 (25%)	9(21%)	17(27%)	*x*^2^(l) = 0.674, *p* = 0412					
Inpatients, *N* (%)^a^^,^^d^	14(14%)	6(14%)	8(14%)	*x*^2^(l) = 0.001, *p* = 0.972					
Age of onset (years)	29.2 ± 9.9	30.8 ± 10.0	28.0 ± 9.7	*t*(104) = 1.432, *p* = 0.155					
Duration of illness (years)	11.5 ± 8.1	11.2 ± 8.3	11.8 ± 8.1	*t*(104) = −0.369, *p* = 713					
History of hospitalization, *N* (%)^a^^,^^e^	41 (40%)	18 (41%)	23 (40%)	*x*^2^(l) = 0.016, *p* = 0.898					
Number of hospitalization^e^	0.95 ± 1.6	0.95 ± 1.5	0.95 ± 1.7	*t*(100) = 0.019, *p* = 0.985					

**Medication use**									
Lithium use, *N* (%)^a^	39 (37%)	23 (52%)	16(26%)	*x*^2^(l) = 7.752, *****p*** = 0.005**					
Valproic acid use, *N* (%)^a^	25 (24%)	8 (18%)	17 (27%)	*x*^2^(l) = 1.219, *p* = 0.270					
Lamotrigine use, *N* (%)^a^	21 (20%)	13 (30%)	8 (13%)	*x*^2^(l) = 4.487, *****p*** = 0.034**					
Antidepressant use, *N* (%)^a^	48 (45%)	24 (55%)	24 (39%)	*x*^2^(l) = 2.605, *p* = 0.107					
Typical antipsychotics use, *N* (%)^a^	14(13%)	4 (9%)	10(16%)	*x*^2^(l) = 1.112, *p* = 0.292					
Atypical antipsychotics use, *N* (%)^a^	42 (40%)	23 (52%)	19 (31%)	*x*^2^(l) = 5.032, *****p*** = 0.025**					
Anxiolytics/hypnotics use, *N* (%)^a^	67 (63%)	31 (71%)	36 (58%)	*x*^2^(l) = 1.699, *p* = 0.192					

**Medication dosage (if any; mg/day)**									
Lithium	587 ± 276	552 ± 292	638 ± 253	*t*(37) = −0.947, *p* = 0.350					
Sodium valproate	476 ± 260	450 ± 302	488 ± 247	*t*(23) = −0.336, *p* = 0.740					
Lamotrigine	157 ± 62	156 ± 72	159 ± 48	*t*(19) = −0.126, *p* = 0.901					
Antidepressant^f^	181 ± 138	192 ± 158	169 ± 116	*t*(45) = 0.579, *p* = 0.565					
Typical antipsychotics^g^	24 ± 22	35 ± 36	20 ± 16	*t*(10) = 1.047, *p* = 0320					
Atypical antipsychotics^g^	179 ± 319	283 ± 276	273 ± 367	*t*(42) = 0.095, *p* = 0.925					

**Symptoms**									
HAM-D21 total score	12.0 ± 8.4	12.2 ± 9.1	11.8 ± 8.0	*t*(104) = 0.242, *p* = 0.809					
YMRS total score^h^	1.7 ± 1.8	2.0 ± 1.7	1.5 ± 1.8	*t*(63) = 1.204, *p* = 0.233					

*^a^Chi-square test was conducted*.

*^b^Information on current smoking status was missing in two (2%) patients and three (1%) controls*.

*^c^Premorbid intelligence quotient (IQ) was estimated from the Japanese Adult Reading Test, which was administered on 100 (94%) subjects with bipolar disorder and all the control subjects*.

*^d^Information was missing in seven (7%) patients*.

*^e^Information on the history of hospitalization was missing in four (4%) patients*.

*^f^Imipramine equivalent for those who received antidepressant medication*.

*^g^Chlorpromazine equivalent for those who received antipsychotic medication*.

*^h^Young Mania Rating Scale (YMRS) data for 19 (43%) male and 22 (35%) female patients were missing*.

*Statistical significance was set at a two-tailed p < 0.05. Bold figures represent significant p value*.

*HAM-D21, 21-item version of the Hamilton Depression Rating Scale; YMRS, Young Mania Rating Scale*.

**Table 2:** Effects of active psychosis and medication use on habituation and PPI percentages (median ± SEM).

	*N*	% Habituation				% PPI_120ms_86dB_				% PPI_120ms_90dB_			
	Yes/no (%)	Yes	No	*U*	*P*	Yes	No	*U*	*P*	Yes	No	*U*	*P*
**Male patients (*n* = 33)**													

Active psychosis^a^	5/28 (15%)	61 ± 8	57 ± 5	56.5	0.498	75 ± 32	59 ± 16	56.0	0.550	75 ± 50	79 ± 11	65.0	0.897
**Medication use**													
Lithium	17/16 (52%)	58 ± 5	57 ± 6	113.5	0.418	57 ± 26	64 ± 8	97.5	0.257	63 ± 21	80 ± 7	111.0	0.533
Sodium valproate	6/27(18%)	55 ± 81	58 ± 5	71.0	0.641	57 ± 34	59 ± 16	59.0	0.659	62 ± 55	79 ± 10	56.5	0.568
Lamotrigine	12/21 (36%)	59 ± 71	58 ± 5	124.5	0.955	68 ± 18	57 ± 20	91.5	0.341	80 ± 26	64 ± 13	94.5	0.404
Antidepressants	21/12 (64%)	58 ± 51	58 ± 5	113.5	0.640	59 ± 20	50 ± 17	104.0	0.648	82 ± 13	62 ± 25	86.0	0.241
Typical antipsychotics	3/30 (9%)	45 ± 18	59 ± 4	17.5	0.085	59 ± 14	59 ± 16	30.0	0.382	83 ± 11	75 ± 13	27.0	0.317
Atypical antipsychotics	16/17 (48%)	57 ± 6	58 ± 5	130.5	0.843	50 ± 28	64 ± 12	92.5	0.186	79 ± 17	75 ± 17	122	0.821
Anxiolytics/hypnotics	24/9 (73%)	57 ± 5	59 ± 6	92.5	0.531	56 ± 19	83 ± 21	67.0	0.126	64 ± 12	83 ± 31	84.0	0.413

**Female patients (*n* = 54)**													

Active psychosis^a^	8/46 (15%)	65 ± 9	75 ± 4	152.0	0.436	25 ± 39	67 ± 8	94.5	**0.029**	−19 ± 100	65 ± 10	103.0	**0.048**
**Medication use**													
Lithium	14/40 (26%)	67 ± 7	74 ± 4	246.5	0.508	54 ± 20	67 ± 11	216.5	0.209	31 ± 41	67 ± 21	238	0.401
Sodium valproate	15/39(28%)	44 ± 8	76 ± 4	187.0	**0.042**	67 ± 19	67 ± 11	267.5	0.628	44 ± 23	63 ± 24	249	0.395
Lamotrigine	6/48(11%)	67 ± 9	74 ± 4	131.0	0.720	50 ± 14	67 ± 11	100.0	0.225	−39 ± 82	62 ± 18	89.0	0.130
Antidepressants	19/35 (35%)	70 ± 7	73 ± 4	307.5	0.651	75 ± 13	57 ± 13	253.0	0.149	66 ± 14	46 ± 28	282.5	0.365
Typical antipsychotics	7/47 (13%)	70 ± 5	73 ± 4	154.0	0.787	50 ± 14	67 ± 11	120.5	0.256	0 ± 69	67 ± 19	77.5	**0.025**
Atypical antipsychotics	16/38 (30%)	61 ± 7	77 ± 4	190.5	**0.031**	71 ± 18	61 ± 11	263.5	0.442	60 ± 22	56 ± 25	288	0.755
Anxiolytics/hypnotics	30/24 (56%)	67 ± 5	78 ± 4	256.5	0.072	67 ± 12	63 ± 15	355.0	0.930	54 ± 22	56 ± 32	352.0	0.889

*^a^Active psychosis was defined as currently having either delusion of guilt, hypochondrias, or paranoia*.

*Group difference was examined with the Mann–Whitney U test*.

*Statistical significance was set at a two-tailed p < 0.05. p-Value was not corrected. Bold figures represent significant p value*.

*PPI_120ms_86dB_, prepulse inhibition (PPI) at a prepuse 120 ms; 86 dB; PPI_120ms_90dB_, PPI at a prepuse 120 ms, 90 dB*.

**Table 3:** Spearman’s correlation of clinical variables with habituation and PPI percentages.

	Habituation		PPI_120ms_86dB_		PPI_120ms_90dB_	
	ρ	*p*-Value	ρ	*p*-Value	ρ	*p*-Value
**Male patients (*n* = 33)**						
Age	0.008	0.967	0.180	0.323	0.168	0.359
Education	0.052	0.774	−0.010	0.959	−0.026	0.889
Premorbid intelligence quotient (IQ) (*n* = 29)	0.325	0.085	−0.325	0.091	−0.256	0.189
Age of onset	−0.089	0.623	0.168	0.359	0.196	0.282
Duration of illness	0.247	0.166	−0.219	0.228	−0.208	0.253
Number of hospitalization	0.002	0.990	0.176	0.335	0.291	0.106
Lithium (if any, *n* = 17)	−0.080	0.761	−0.195	0.452	0.304	0.235
Sodium valproate (if any, *n* = 6)	0.759	0.080	−0.671	0.215	−0.447	0.450
Lamotrigine (if any, *n* = 12)	−0.066	0.840	0.594	0.054	0.813	**0.002**
Antipsychotics (if any, *n* = 16)^a^	−0.056	0.836	−0.171	0.543	0.086	0.762
Typical antipsychotics (*n* = 2)	**–**	**–**	**–**	**–**	**–**	**–**
Atypical antipsychotics (*n* = 16)	−0.015	0.957	−0.257	0.354	0.007	0.980
Antidepressant (if any, *n* = 21)^b^	−0.080	0.731	−0.113	0.625	0.118	0.611
HAM-D21 total	0.077	0.668	−0.309	0.086	−0.240	0.186
YMRS total (*n* = 19)	0.145	0.554	0.267	0.284	0.336	0.172

**Female patients (*n* = 54)**						
Age	−0.070	0.62	−0.084	0.54	0.060	0.666
Education	−0.025	0.857	0.076	0.586	0.153	0.269
Premorid IQ (*n* = 52)	0.027	0.847	0.193	0.171	0.207	0.141
Age of onset	0.081	0.564	−0.108	0.440	0.055	0.696
Duration of illness	0.021	0.878	−0.061	0.659	−0.003	0.982
Number of hospitalization (*n* = 50)	−0.131	0.365	0.113	0.435	0.117	0.419
Lithium (if any, *n* = 14)	**−0.570**	**0.033**	**0.691**	**0.006**	0.465	0.094
Sodium valproate (if any, *n* = 15)	0.070	0.805	0.101	0.720	0.136	0.629
Lamotrigine (if any, *n* = 6)	0.304	0.558	−0.257	0.623	−0.101	0.848
Antipsychotics (if any, *n* = 21)^a^	−0.118	0.611	−0.028	0.904	0.061	0.794
Typical antipsychotics (*n* = 6)	0.257	0.623	0.600	0.208	0.371	0.468
Atypical antipsychotics (*n* = 18)	−0.220	0.380	−0.185	0.463	−0.100	0.694
Antidepressant (if any, *n* = 19)^b^	−0.193	0.427	0.309	0.199	0.202	0.407
HAM-D21 total	−0.017	0.906	−0.143	0.303	−0.253	0.065
YMRS total (*n* = 34)	−0.044	0.806	0.010	0.957	−0.086	0.631

*^a^Chlorpromazine equivalent for those who received antipsychotic medication*.

*^b^Imipramine equivalent for those who received antidepressant medication*.

*Statistical significance was set at a two-tailed p < 0.05. Bold figures represent statistical significance*.

*HAM-D21, 21-item version of the Hamilton depression rating scale; YMRS, Young Mania Rating Scale; PPI_120ms_86dB_, prepulse inhibition (PPI) at a prepuse 120 ms, 86 dB; PPI_120ms_90dB_, PPI at a prepuse 120 ms, 90 dB*.

### Startle Reflex and Habituation

Neither BSR magnitude nor habituation percentage significantly differed between the two diagnostic groups or among the three clinical groups. Neither BSR nor habituation correlated with the total HAM-D21 or YMRS, respectively. BSR negatively correlated with age in male patients (ρ = −0.481, *p* = 0.001), but significance disappeared when non-responders were excluded (ρ = −0.270, *p* = 0.129). Female patients medicated with sodium valproate and/or atypical antipsychotics exhibited significantly lower habituation than did those without such medication. Habituation in female patients was significantly negatively correlated with lithium dosage (ρ = −0.570, *p* = 0.033). No other association with habituation was found.

### Prepulse Inhibition

#### Comparisons Between Clinical Groups

Male patients showed significantly lower PPI than male controls at PPI_120ms_86dB_ (*U* = 913.0, *p* = 0.034); however, female patients did not show a significant difference from female controls at any PP condition. Stratified analysis across the three clinical groups detected significantly reduced PPI in depressed male patients compared to male controls at PPI_120ms_86dB_ [χ^2^(2) = 6.456, *p* = 0.040], while there were no statistically significant differences between euthymic male patients and male controls or between euthymic and depressed male patients. PPI in female patients did not differ from that in female controls either in the depressed or the euthymic state. The results were virtually the same when we excluded patients with concurrent psychiatric disorders (Table S3 in Supplementary Material). When we examined the incidence of low-PPI (first quartile of controls) vs. high-PPI (second to fourth quartiles) across the three clinical groups, there was a significantly higher incidence of low-PPI among male patients with depression (55%) compared to their respective controls (*p* = 0.039; Figure 3; Table S2 in Supplementary Material). There was also a trend for a higher incidence of low-PPI in male patients with depression compared to euthymic male patients (*p* = 0.070).

#### Effects of Active Psychosis and Medication Status on PPI

Female patients with active psychosis showed significantly lower PPI than did those without psychosis. Female patients on typical antipsychotics had significantly lower PPI than those without such medication (Table 2).

#### Correlation of Clinical Variables With PPI

There was a trend for HAM-D21 total to correlate negatively with PPI in male (ρ = −0.309, *p* = 0.086 at PPI_120ms_86dB_) and female patients (ρ = −0.253, *p* = 0.065 at PPI_120ms_90dB_). YMRS score was not correlated with PPI. PPI was significantly positively correlated with lamotrigine dosage in male patients (ρ = 0.813, *p* = 0.002 at PPI_120ms_90dB_), and lithium dosage in female patients (ρ = 0.691, *p* = 0.006 at PPI_120ms_86dB_) (Table 3). No other association with PPI was found.

## Discussion

To our knowledge, this is the largest PPI study in BD patients (*n* = 106), and the first study exploring PPI in BD patients with depression (*n* = 63). The large sample size enabled us to conduct the analyses stratified by gender and state (depressed/euthymic). This study aimed to clarify how a state (i.e., depressed/euthymic) is associated with PPI in non-manic BD patients. Our main findings are as follows. First, as hypothesized, male patients with BD, but not female patients, had significantly lower PPI than male controls at one of the PP conditions (PPI_120ms_86dB_). More specifically, male patients with depression, but not euthymic male patients, had significantly lower PPI than male controls. More than half of the male patients with depression had low-PPI (< first quartile of male controls). In contrast, PPI in female patients did not differ from that in female controls, either in the depressed or the euthymic state. Female patients with active psychosis showed significantly lower PPI than those without psychosis. Female patients on typical antipsychotics had significantly lower PPI than those without such medication. PPI was significantly positively correlated with lamotrigine dosage in male patients and lithium dosage in female patients.

Few studies have investigated PPI in psychiatric disorders separately by gender and most reported that PPI deficits were found only in male patients, such as those with chronic schizophrenia (74), euthymic BD (45), and MDD (53). The present finding is in line with those studies but it contradicts with our previous study which found PPI deficits both in men and women with schizophrenia (6). The seemingly intact PPI in female BD in the present study is in line with the above report on euthymic BD by Gogos et al. (45), which tested female subjects who were all in the follicular phase and reported that PPI in female BD was significantly higher than that in female controls. Our study did not control the time of testing in the menstrual cycle; however, we have previously obtained similar results of seemingly intact PPI in female MDD and speculated that possible menstrual irregularity in some female patients caused by psychotropic medication, insomnia, and psychological stress might have increased PPI in female patients (53). Future studies on women should be made in the follicular phase to minimize the effects of circulating ovarian hormones.

Our finding of impaired PPI in male BD patients with depression and seemingly intact PPI in euthymic males with BD suggests that PPI is state dependent in men with BD. We also found a trend for the HAM-D21 total to correlate negatively with PPI in male and female BD. However, since the number of euthymic male BD is limited (*n* = 13), the above finding may have the risk of type II error. Gogos et al. (45) reported the presence of PPI deficits in the euthymic male with BD; however, half of their male patients (10/18) had mild to severe depression and, therefore, may not be described as “euthymic” (45). Other studies which examined PPI in euthymic BD in an equal mix of men and women found inconsistent results: two studies found normal PPI (35, 36), whereas the other two studies reported significant PPI deficits in euthymic BD (37, 38). A possible reason for such inconsistency may be that the latter two studies tested only patients with BD I, whereas there were only two euthymic and six depressed patients with BD I among the responders in our study. We conducted a sensitivity analysis excluding male patients with BD I and found that the results were virtually unchanged (median PPI_120ms_86dB_ was 66% in 11 euthymic male patients and 48% in 14 depressed male patients). Again, we found significant differences between depressed BD II patients and controls (*U* = 301.0, *p* = 0.009) and between depressed BD II patients and euthymic BD II patients (*U* = 36.0, *p* = 0.048), but no significant difference between euthymic BD II patients and controls, in men. Taken together, PPI in patients with BD II may be state dependent. To address this issue, further longitudinal studies are required.

Against our second hypothesis, we were unable to find a significant correlation between startle measurements (habituation and PPI) and variables related to the severity of psychopathology, such as the age of illness onset, duration of illness, and the number of hospitalization. Barret et al. found a significant correlation between PPI and these clinical variables, concluding that an early onset of illness has a detrimental effect on PPI levels (35). Gogos et al. also found a trend for a correlation between PPI and age of onset in male BD patients (45). Our finding that female BD patients with current psychosis had a significantly lower PPI than those without such psychosis is in line with the previous literature indicating the association of PPI deficits with the presence of psychosis and thought disorder (20, 33, 34). However, psychosis might not be a key determinant, considering that we were unable to find such an association in males.

Psychotropic medication may be a possible confounding factor that may have masked the direct association of depression with PPI. In the present study, we obtained tentative evidence of deteriorating effects of sodium valproate and/or atypical antipsychotics on habituation and typical antipsychotics on PPI in female patients. Some studies suggest that atypical antipsychotics may improve PPI deficits in schizophrenia (64); however, we were unable to find such an effect in BD patients. The imbalance in monoaminergic neurotransmission, changes in the activity of monoamine transporters, hyper- and hypo-dopaminergic function, and imbalance of excitatory/inhibitory neurotransmission by glutamate and γ-aminobutyric acid (GAVA) systems have been posited as the neurobiological hypotheses of BD (30). In the present study, neither patients medicated with antipsychotics, antidepressants, nor mood stabilizers showed better PPI than those who were not medicated. On the other hand, we found a strong positive correlation of PPI with lamotrigine dosage in male and lithium in female patients. These findings may suggest that lamotrigine and lithium ameliorate the PPI deficits if sufficient dosage is prescribed. Our finding is in line with the previous studies reporting that lamotrigine and lithium were superior to placebo for the prevention of mood episodes in patients with BD I (75, 76). Lithium is suggested to increase inhibitory neurotransmission based on the finding that GAVA levels were increased after chronic lithium treatment (77). Alternatively, the results may have arisen by chance. Since most of the patients were treated by combined medication, interpretation of specific effects of each drug should be made with caution. Previous literature of the comparative trials on the effects of sodium valproate, haloperidol, aripiprazole, or other antipsychotics on BD patients are still limited (78–81). Some studies reported the potential ameliorating effect of a low dose of the NMDA receptor antagonist ketamine (3, 82) and l-theanine (*N*-ethyl-l-glutamine), a component of green tea, on PPI (83, 84). These findings may support a new treatment strategy on gene expression changes in glutamatergic neurotransmission and GluR expression commonly identified among schizophrenia, BD, and MDD by GWAS, postmortem brain, and magnetic resonance spectroscopy studies (29–32).

This study includes the following limitations. First, this is a cross-sectional study. Longitudinal intra-individual studies are needed to confirm the present findings and examine whether the reduced PPI in BD patients with depression is normalized in remission. Second, our results include the effects of medication. Third, the number of patients with BD I in the present study is limited (17 depressed, 9 euthymic; 25% of total patients with BD), and therefore, we were unable to examine PPI deficits exclusively in patients with BD I. Finally, we did not collect information about the history of psychosis. Although we found no correlation of startle measurements with psychopathological severity such as the age of illness onset, duration of illness, and the number of hospitalization, we were unable to analyze the effect of psychotic episodes.

In conclusion, our findings suggest that sensorimotor gating is impaired in male BD patients with depression. However, we obtained no evidence for such abnormalities in female BD patients except for those with current psychosis. The observed associations between medication and startle measurements warrant further investigation.

## Ethics Statement

This study was performed in accordance with the Declaration of Helsinki and approved by the Ethics Committee of the National Center of Neurology and Psychiatry, Japan. Written informed consent for participation in this study was obtained from every subject.

## Author Contributions

JM made statistical analysis, managed literature search, interpreted the data, and wrote the draft of the manuscript. MO and SH analyzed the EMG data. MO, SH, TT, and HH conducted clinical interviews. JM, II, and MH contributed for data collection. HK supervised the entire project and gave critical comments on the manuscript. All authors contributed substantially to this work and had approved the final manuscript.

## Conflict of Interest Statement

The authors have declared that the research was conducted in the absence of any commercial or financial relationships that could be construed as a potential conflict of interest.
